# In vitro susceptibilities of *Brucella melitensis *isolates to eleven antibiotics

**DOI:** 10.1186/1476-0711-5-24

**Published:** 2006-10-02

**Authors:** Aun Turkmani, Alexandros Ioannidis, Athanasia Christidou, Anna Psaroulaki, Feidias Loukaides, Yiannis Tselentis

**Affiliations:** 1 Department of Clinical Bacteriology, Parasitology, Zoonoses, and Geographical Medicine, Faculty of Medicine, University of Crete, Greece; 2 Veterinary services of Cyprus, Athalassa, Nicosia, Cyprus

## Abstract

**Background:**

Brucellosis is an endemic disease present in many countries worldwide, but it is rare in Europe and North America. Nevertheless brucella is included in the bacteria potentially used for bioterrorism. The aim of this study was the investigation of the antibiotic susceptibility profile of brucella isolates from areas of the eastern Mediterranean where it has been endemic.

**Methods:**

The susceptibilities of 74 Brucella melitensis isolates derived from clinical samples (57) and animal products (17) were tested in vitro. The strains originate from Crete (59), Cyprus (10), and Syria (5). MICs of tetracycline, rifampicin, streptomycin, gentamicin, norfloxacin, ciprofloxacin, levofloxacin, trimethoprim/sulfamethoxazole, ampicillin, amoxicillin/clavulanic acid, and erythromycin were detected by E-test method. The NCCLS criteria for slow growing bacteria were considered to interpret the results.

**Results:**

All the isolates were susceptible to tetracycline, streptomycin, gentamicin, ciprofloxacin, norfloxacin, and levofloxacin. Two isolates presented reduced susceptibility to rifampicin (MIC value: 1.5 mg/l) and eight to SXT (MIC values: 0.75–1.5 mg/l). Erythromycin had the highest (4 mg/l) MIC_90_value and both norfloxacin and erythromycin the highest (1.5 mg/l) MIC_50 _value.

**Conclusion:**

Brucella isolates remain susceptible in vitro to most antibiotics used for treatment of brucellosis. The establishment of a standardized antibiotic susceptibility method for *Brucella spp *would be useful for resistance determination in these bacteria and possible evaluation of bioterorism risks.

## Background

*Brucella *is a pathogen of global dispersal, which affects both humans and animals. This dispersal has exhibited a shift towards the Asian countries; on the contrary there is a significant reduction in brucellosis cases, in countries where brucellosis was endemic in the past such as Spain and Italy [1]. At the moment brucellosis is scarce in Western Europe and North America since effective public health measures have been established, however such countries are considering Brucella to be a potential bioterrorism threat leading to an increased interest in those countries [2].

*Brucella *is an intracellular bacterial pathogen that infects host macrophage cells. In consequence, specialized agents that are able to penetrate the macrophages and function within their cytoplasm are required for the treatment of brucellosis. Tetracyclines, rifampicin, trimethoprim-sulphamethoxazole (SXT), streptomycin, and other aminoglycosides, separately or in combinations, are most commonly used for brucellosis treatment [3,4]. Fluoroquinolones, and macrolides may serve as an alternative drug choice [5,6]. In 1986, the WHO has released recommendations for use of doxycycline, combined with either rifampicin or streptomycin for treating human brucellosis. This recommendation is still in function.

*Brucella *isolates are generally considered susceptible to the recommended by the WHO antibiotics. Nevertheless sporadic cases of a kind of antibiotic resistance have been reported [7,8]. The aim of this essay was to determine the antibiotic susceptibilities of the *Brucella *isolates identified in the Laboratory of Clinical Bacteriology, Parasitology, Zoonoses, and Geographical medicine of the University of Crete.

## Methods

A number of 74 strains were included in this research both of human and animal origin. The human samples were cultured using the automated blood culture system (Organon Teknika BacT/Alert, Biomerieux^®^, France) and the animal strains were isolated using Brucella selective culture media. Typing of all *Brucella *isolates was based on conventional microbiological methods (the requirement of CO_2 _for growth, production of urease and H_2_S, sensitivity to the dyes basic fuchsin and thionin, and lysis by the phages Tiblissi and Weybridge).

In addition to the 74 strains, 6 *Brucella *reference strains: *B. abortus *104 M, *B. abortus *2308, *B. melitensis *16 M biotype 1 (ATCC 23456), *B. abortus *B3196 biotype 5 (ATCC 23452), *B. suis *513 biotype 5, and *B. neotomae *5K33 (ATCC 23459) were also tested. The reference strains: *Esherichia coli *ATCC 25922, *Staphylococcus aureus *ATCC 29213 were used as control.

Antibiotic susceptibility testing was performed by the E-test method (AB biodisk) according to the manufacturer's guidelines. The 11 antibiotics tested were: tetracycline, rifampicin, streptomycin, gentamicin, norfloxacin, ciprofloxacin, levofloxacin, trimethoprim/sulfamethoxazole, ampicillin, amoxicillin/clavulanic acid, and erythromycin. The bacterial suspension, in a concentration equal to 10^5 ^– 10^6 ^cfu/ml, was inoculated on Mueller Hilton agar plates, supplemented with 5% sheep blood agar, and the E- test strips were applied. The plates were incubated at 35°C in a 5% CO_2 _atmosphere for 48 h.

The NCCLS interpretive criteria for slow growing bacteria (Haemophilus) were also taken into consideration in order to evaluate the results of MICs determination [9].

## Results

The analysis of the data concerning the isolates resulted in the following. All isolates are from the time period 1999–2005 and originate from various regions of the Eastern Mediterranean (Crete, 59; Cyprus, 10; and Syria, 5).

Seventeen isolates were obtained from sick animals (sheep and goats); the source of isolation was animal products, mainly milk. The remaining 57 isolates originate from patients (Table 1). Amongst the 55 patients from Crete, 5 were immigrants from the Balkan area, recently immigrated and were therefore possibly infected in their prior settlements. In additional, four of the patients were in relapse while the rest were in acute phase. The clinical isolates were obtained from blood (44), bone marrow (3), synovial fluid (2), cerebrospinal fluid (1), bone tissue (2), and juxtaspinal abscess (1).

**Table 1:** Distribution of human and animal derived *Brucella melitensis *isolates by geographical location

Geographical Location	Human Origin (No)	Animal Origin (No)	Total (No)
Crete (Greece)	55	4	59
Cyprus	2	8	10
Syria		5	5
Total	57	17	74

All isolates were identified as *Brucella melitensis*.

The MIC_50 _and MIC_90 _values of the antibiotics are shown in Table 2. The MIC values of tetracycline, ciprofloxacin, levofloxacin, and amoxicillin/clavulanic acid, interpreted according to the NCCLS criteria for slow growing bacteria, have shown ranges below the breakpoints for sensitivity determination. The MIC values of ampicillin, rifampicin, and SXT range at levels below the breakpoints for resistance determination. The MIC of rifampicin is 1.5 mg/l for two isolates and the MIC values of SXT range from 0.75 mg/l to 1.5 mg/l for eight of the isolates. The MICs of streptomycin and gentamicin were also low. The erythromycin among the total of the antibiotics and the norfloxacin among the quinolones presented the highest MIC values.

**Table 2:** In vitro susceptibilities of *Brucella melitensis *isolates to 11 antibiotics

Antibiotics	Range (mg/l)	MIC50 (mg/l)	MIC90 (mg/l)
**Tetracycline**	0.032 – 1.5	0.125	0.5
**Rifampicin**	0.094 – 1.5	0.5	1
**Streptomycin**	0.125 – 4	1	2
**Gentamicin**	0.032–1.5	0.19	2
**Norfloxacin**	0.125 – 4	1.5	3
**Ciprofloxacin**	0.016 – 0.75	0.19	0.5
**Levofloxacin**	0.064 – 0.75	0.25	0.5
**Trimethoprim/sulfamethoxazole**	0.032/0.61 – 1.5/28.5	0.125/2.38	0.75/14.2
**Ampicillin**	0.094 – 3	0.75	2
**Amoxicillin/clavulanic acid**	0.032 – 1.5	0.38	1
**Erythromycin**	0.5 – 8	1.5	4

Furthermore, no kind of resistance was detected in the isolates derived from patients in relapse and their MIC values were at low levels.

## Discussion

*Brucella species *are highly infectious pathogens and level 3 biosafety precautions must be kept during the susceptibility testing procedure. These pathogens are considered to be susceptible to the antibiotics recommended by the WHO for treatment of brucellosis. Subsequently, Brucella susceptibility testing is not routinely performed. Additionally, there is no standardized method for susceptibility testing recommended by NCCLS for these microorganisms. Relapses, at a rate of about 10 percent, usually occur in the first year after the infection, but they are caused by inadequate treatment in most cases [3]. Antibiotic-resistant *Brucella *strains are rarely a cause of therapy failure [4]. However, strains resistant to the main antimicrobial agents may emerge [10] and lead on to treatment inhibition.

There are few reports for the in vitro susceptibilities of *Brucella *and various methodologies have been applied. Broth microdilution [11-13], agar dilution [5,14], and E-test methods [7,13,15] have been applied for antibiotic MIC determinations. Brucella agar [11], Muller-Hinton agar, and Muller-Hinton broth supplemented with 1% Polyvitex [12-14], or combined 1% Polyvitex and 1% haemoglobin [5], and Muller-Hinton agar supplemented with 5% sheep blood agar [7,13] are the media used for antibiotic susceptibility testing of *Brucella*. E-test is a reliable, reproducible, and easily performed method for antimicrobial susceptibility testing and has been successfully employed for the testing of *Brucella *strains [7,13,14].

The isolates included in this study, originated from three Eastern Mediterranean countries (Greece, Cyprus, and Syria) and were highly susceptible to most antibiotics tested, which is consistent with previous reports. Tetracycline (MIC90: 0.5 mg/l) was proved to be active in vitro against all the isolates; this finding agrees with previous reports [7,11,14,15]. Rifampicin (MIC90: 1) also exhibited good activity. However two of the total (74) isolates were inhibited by 1.5 mg/l of rifampicin. Since breakpoints have not yet been established for *Brucella species*, these strains cannot be confidently characterised as of intermediate resistance, although a reduced susceptibility may exist. MIC values of rifampicin ranging from 1 to 4 mg/l have already been reported [7,8]. A similar result was obtained for SXT. Eight of the 74 isolates with MIC values from 0.75 to 1.5 mg/l may be characterized as intermediate resistance strains according to the NCCLS interpretive criteria for slow growing bacteria. Significant rates of SXT resistance have been reported in previous studies [7,8].

MICs of aminoglycosides (streptomycin, and gentamicin) were low, corresponding to in vitro susceptibility of all isolates, which is consistent with previous reports [6,11].

Several studies focused on quinolones activity against *Brucella*, because these agents appeared as an attractive alternative drug choice for human brucellosis treatment. Although in vitro resistance to quinolones is not high, the effectiveness of these antibiotics remains controversial [5,6,11,12]. Our isolates were inhibited in vitro by low concentrations of quinolones.

The role of macrolides in brucellosis treatment also remains controversial [5,6]. MIC values of erythromycin ranged from 0.5 to 8 mg/l, indicating reduced activity. Erythromycin, ampicillin, and amoxicillin/clavulanic acid were included in the study for research purposes only, as those agents are ineffective in vivo for brucellosis treatment. Subsequently, the low MIC values of ampicillin and amoxicillin/clavulanic acid found in our isolates do not correspond to any therapeutic effect.

## Conclusion

Brucellosis remains a major public health problem in countries with low socialeconomical status. The necessity to keep rifampicin for tuberculosis treatment and the requirement of alternative drug therapy for specialized cases entails the research for other antibiotic usage. Subsequently, the antibiotic susceptibility testing of *Brucella *may help the choice of treatment in specific cases, the epidemiological surveys and the prediction pottential threats. Therefore, the establishment of a simple, reliable, and low-costing method for *Brucella *susceptibility testing would be useful for an early detection of any drug resistance that may be developed.

## Competing interests

The author(s) declare that they have no competing interests.

## Authors' contributions

AT carried out the acquisition, analysis and interpretation of the data. AI performed the computation analysis of the study and critically revised the manuscript. AC performed the analysis and interpretation of the data and drafted the manuscript. FL contributed in acquisition of data. AP and YT carried out the design and coordination of the study. All authors read and approved the final manuscript.

## References

[B1] Pappas G, Papadimitriou P, Akritidis N, Christou L, Tsianos EV (2006). The new global map of human brucellosis. Lancet Infect Dis.

[B2] Bossi P, Tegnell A, Baka A, Van Loock F, Hendriks J, Werner A, Maidhof H, Gouvras G, Task Force on Biological and Chemical Agents Threats, Public Health Directorate, European Commission, Luxembourg (2004). Bichat guidelines for the clinical management of brucellosis and bioterrorism-related brucellosis. Euro Surveill.

[B3] Pappas G, Akritidis N, Bosilkovski M, Tsianos E (2005). Brucellosis. N Engl J Med.

[B4] Hall WH (1991). Modern chemotherapy for brucellosis in humans. Rev Infect Dis.

[B5] Garcia-Rodriguez JA, Garcia-Sanchez JE, Trujillano I (1991). Lack of effective bactericidal activity of new quinolones against Brucella spp. Antimicrob Agents Chemother.

[B6] Qadri SM, Halim MA, Ueno Y, Abumustafa FM, Postle AG (1995). Antibacterial activity of azithromycin against Brucella melitensis. Chemotherapy.

[B7] Baykam N, Esener H, Ergonul O, Eren S, Celikbas AK, Dokuzoguz B (2004). In vitro antimicrobial susceptibility of *Brucella *species. Intern J Antimicrob Agents.

[B8] Lopez-Merino A, Contreras-Rodriguez A, Migranas-Ortiz R, Orrantia-Gradin R, Hernandez-Oliva GM, Guttierrez-Rubio AT, Cardenosa O (2004). Susceptibility of Mexican brucella isolates to moxifloxacin, ciprofloxacin and other antimicrobials used in the treatment of human brucellosis. Scand J Infect Dis.

[B9] National Committee for Clinical Laboratory Standards (1998). Performance standards for antimicrobial susceptibility testing. Eighth informational supplement NCCLS document M 100-S8.

[B10] Marianelli C, Ciuchini F, Tarantino M, Pasquali P, Adone R (2004). Genetic bases of the rifampin resistance phenotype in *Brucella spp*. J Clin Microbiol.

[B11] Rubinstein E, Lang R, Shasha B, Hagar B, Diamanstein L, Joseph G, Anderson M, Harrison K (1991). In vitro susceptibility of Brucella melitensis to antibiotics. Antimicrob Agents Chemother.

[B12] Akova M, Uzun O, Akalin HE, Hayran M, Unal S, Gur D (1993). Quinolones in the treatment of human brucellosis: comparative trial of ofloxacin-rifampin versus doxycycline-rifampin. J Antimicrob Chemother.

[B13] Gur D, Kocagoz S, Akova M, Unal S (1999). Comparison of E test to Microdilution for determining of in vitro activities of antibioticsagainst *Brucella melitensis*. Antimicrob Agents Chemother.

[B14] Yamazham T, Aydemir S, Tunger A, Serter D, Gokengin D (2005). Invitro activities of various antimicrobials against *Brucella melitensis *strains in the Aegean region in Turkey. Med Princ Pract.

[B15] Bodur H, Balaban N, Aksaray S, Yetener V, Akinci E, Colpan A, Erbay A (2003). Biotypes and antimicrobial susceptibilities of Brucella isolates. Scand J Infect Dis.

[B16] Al Dakour S, Hagen RM, Nockler K, Tomaso H, Witting M, Scholz HC, Vergnaud G, Neubauer H (2005). Failure of a short-term antibiotic therapy for human brucellosis using ciprofloxacin. A study on in vitro susceptibility of Brucella strains. Chemotherapy.

